# Characterization, antioxidant activity, and mineral profiling of *Auricularia cornea* mushroom strains

**DOI:** 10.3389/fnut.2023.1167805

**Published:** 2023-06-19

**Authors:** Asif Ali Khan, Li-Xin Lu, Fang-Jie Yao, Ming Fang, Peng Wang, You-Min Zhang, Jing-Jing Meng, Xiao-Xu Ma, Qi He, Kai-Sheng Shao, Yun-hui Wei, Baojun Xu

**Affiliations:** ^1^College of Horticulture, Jilin Agricultural University, Changchun, China; ^2^International Cooperation Research Center of China for New Germplasm Breeding of Edible Mushrooms, Jilin Agricultural University, Changchun, China; ^3^Institute of Economical Plants Research, Academy of Agricultural Science of Jilin Province, Gongzhuling, China; ^4^College of Forestry and Grassland, Jilin Agricultural University, Changchun, China; ^5^Jiangxi Academy of Agricultural Sciences Nanchang, Nanchang, China; ^6^Food Science and Technology Program, Department of Life Sciences, BNU-HKBU United International College, Zhuhai, China

**Keywords:** *Auricularia cornea*, wild mushroom, anti-oxidant activity, minerals, polysaccharides

## Abstract

**Background:**

Mushrooms are considered as next-generation healthy food components. Owing to their low-fat content, high-quality proteins, dietary fiber, and rich source of nutraceuticals. They are ideally preferred in formulation of low-caloric functional foods. In this view, the breeding strategies of mushroom *Auricularia cornea* (*A. cornea*) focusing on high yield and higher quality with rich nutritional values and health benefits are still needed.

**Materials and methods:**

A total of 50 strains of *A. cornea* were used to analyze the bio efficiency and the time required for fruiting body formation following the cultivation experiment. The calorimetric method was used to evaluate the antioxidant activity and quantify the crude polysaccharides and minerals content thereafter.

**Results:**

The results showed that the time required for fruiting body formation and biological efficiency varied significantly among the selected strains. Noticeably, the wild domesticated strain Ac13 of *A. cornea* mushroom showed the shortest fruit development time (80 days). Similarly, the hybrid strains including Ac3 and Ac15 possessed the highest biological efficiency (82.40 and 94.84%). Hybrid strains Ac18 (15.2%) and cultivated strains Ac33 (15.6%) showed the highest content of crude polysaccharides, while cultivated strains Ac1 and Ac33, demonstrated the highest content of total polysaccharides in the fruiting body (216 mg. g^−1^ and 200 mg. g^−1^). In the case of mineral content, the highest zinc contents were observed from the cultivated strain Ac46 (486.33 mg·kg^−1^). The maximum iron content was detected from the hybrid strain Ac3 (788 mg·kg^−1^), and the wild domesticated strain Ac28 (350 mg·kg^−1^). The crude polysaccharides of the *A. cornea* strain showed significant antioxidant potential, and the ability of Ac33 and Ac24 to scavenge DPPH radicals and ABTS, which was significantly improved compared to other strains, respectively. Principal component analysis was applied to examine the agronomic traits and chemical compounds of various strains of *A. cornea* mushrooms. The results revealed that cultivated, wild domesticated, and hybrid strains of *A. cornea* exhibited distinct characteristics in terms of growth, yield, and nutritional properties.

**Conclusion:**

The crude polysaccharides from *A. cornea* mushroom strains act as natural antioxidants, the wild, hybrid, and commercial *A. cornea* mushroom strains can achieve rapid growth, early maturation, and high yields. The evaluation of biochemical indexes and nutritional characteristics of strains with excellent traits provided a scientific basis for initiating high-quality breeding, provided germplasm resources for the production of “functional food” with real nutritional and health value.

## Introduction

Mushrooms are the only treasure trove of biologically active metabolites that are extremely rich in high-quality carbohydrates, minerals, proteins, and dietary fiber (1, 2). Under the current world scenario, people eagerly await novel natural, non-toxic functional foods that provide nutritional, and medicinal value (3). With their unique aroma, mushrooms become a delicious food, a step ahead of other natural resources.

Mushroom polysaccharides are considered suitable biological response regulators because of their wide range of health benefits (4, 5). Among all mushroom-derived substances, polysaccharides have been shown to have potential biological activities, including anti-inflammatory, antioxidant, anticancer, antitumor, and significant effects on innate and adaptive immunity (6). One of the effective bioactive compounds of edible fungi is polysaccharides, and another important feature of mushroom polysaccharides is their potential to scavenge free radicals (7, 8). Prior studies revealed that free radicals are the key factor behind several chronic diseases, including neurological disorders, cancer, arthritis, diabetes, and others (9). Although many synthetic antioxidants such as butylated hydroxyanisole, tert-butyled hydroxyquinine, and butylated hydroxytoluene are readily available in the market, the main concerns are increasing about the safety of these synthetic products used in conventional foods. The regular intake of a substance has been found to have detrimental effects on the health of individuals, and prolonged utilization may result in severe adverse reactions. In this regard, mushroom polysaccharides may be recommended as a good alternative to the food industry as a novel potential antioxidant and low-toxicity alternative.

*Auricularia cornea* is a medicinal and edible mushroom. It belongs to the fungi Basida, Auriculariaceae, mainly distributed in China, Korea and other East Asian countries (10). *Auricularia* is the fourth most widely cultivated mushroom worldwide and a popular ingredient in Chinese cuisine and medicine. Fungus is one of the most widely cultivated mushrooms in China (11). According to the China Edible Mushroom Association, the annual production of *A. auricularia* and *A. cornea* reached 75.2 million tons and 16.9 million tons, respectively, in 2017 (12). It is also cultivated in other parts of the world. The advantage of *A. cornea* mushroom is its long shelf life (13). Due to its ability to undergo desiccation, this particular species presents a viable option for growers seeking to propagate mushrooms in contrast to alternative varieties (14). *A. cornea* is a low-calorie food that is rich in nutrients including good source of complex carbohydrates, low-fat source of protein, B vitamins, including riboflavin, niacin, and pantothenic acid, minerals such as iron, potassium, and phosphorus, polysaccharides, and consumed in moderation as part of a balanced diet (11). Though the determination of amino acids is important for assessing the nutritional value of a food or dietary supplement, it may not always be necessary in studies of *A. cornea* as it is already been shown to be a good source of protein, containing all essential amino acids (15). Therefore, we focused on other aspects of its nutritional composition, such as its mineral content, carbohydrate content, or antioxidant activity, rather than determining the amino acid profile. The mineral content and antioxidants of *A. cornea* can help determine its potential as a dietary supplement for maintaining optimal health, reduction of oxidative stress and inflammation in the body. However, the polysaccharides, minerals, and antioxidant properties of *A. cornea* have not been studied using many germplasm resources. Therefore, this study focused on the time required for fruiting body formation, biological efficiency, polysaccharide content, antioxidant activity, and mineral element content of 50 *A. cornea* mushroom strains from China and Japan and newly developed *A. cornea* varieties.

## Materials and methods

### Mushroom strains

In this study, a total of 50 strains of *A. cornea* were used (listed in Table 1). Among these strains, 14 commercial cultivated strains were obtained from distinct regions of China, namely Sichuan Academy of Agricultural Sciences, Zhangzhou Comprehensive Experimental Station, College of Horticulture at Jilin Agricultural University, as well as from Japan. Furthermore, the study also included 18 wild *A. cornea* strains from five different provinces in China, namely Jiaohe City, Jilin Province; Yitong City, Jilin Province; Chengzijie Town, Jiutai City, Jilin Province, Jilin Agricultural University Campus, Changchun City, Jilin Province, Xiamafang Ruins Park, Nanjing City, Jiangsu Province; East bank of West Lake, Hangzhou City, Zhejiang Province; Dashushan Forest Park, Hefei City, Anhui Province; Tongbai County Buddhist College, Henan Province, as well as strains from Japan. In addition, the study involved 14 hybrid *A. cornea* strains.

**Table 1:** List of *Auricularia cornea* strains.

Strains	Name of strains	Source of strains
AC1	Ya	College of Horticulture, Jilin Agricultural University (Cultivated Strains)
AC2	Ap18	Sichuan Academy of Agricultural Sciences (Cultivated Strains)
AC3	Ac3	Hybrid strain
AC4	Ap17	Sichuan Academy of Agricultural Sciences (Cultivated Strains)
AC5	Ac5	Hybrid strain
AC6	Ac6	Hybrid strain
AC7	Ac7	Hybrid strain
AC8	Ac8	Hybrid strain
AC9	Ac9	Hybrid strain
AC10	Ac10	Hybrid strain
AC11	Ac11	Hybrid strain
AC12	Ac12	Hybrid strain
AC13	TB	Paulownia tree (wild strain), Tongbai County Buddhist College, Henan Province
AC14	Ac14	Hybrid strain
AC15	Ac15	Hybrid strain
AC16	Ac16	Hybrid strain
AC17	Ac17	Hybrid strain
AC18	Ac18	Hybrid strain
AC19	Ac19	Hybrid strain
AC20	Ac20	Hybrid strain
AC21	Ac21	Hybrid strain
AC22	A406	Chinese tallow tree (wild strain), Dashu Mountain Forest Park, Hefei, Anhui
AC23	A407	Chinese tallow tree (wild strain), Dashu Mountain Forest Park, Hefei, Anhui
AC24	A408	Chinese tallow tree (wild strain), Dashu Mountain Forest Park, Hefei, Anhui
AC25	A409	Chinese tallow tree (wild strain), Dashu Mountain Forest Park, Hefei, Anhui
AC26	A412	Plane tree (wild strain) on the east bank of West Lake, Hangzhou City, Zhejiang Province
AC27	Ac27	(wild strain) Hangzhou City, Zhejiang Province
AC28	A414	Plane tree (wild strain) in Xiamafang Ruins Park, Nanjing City, Jiangsu Province
AC29	A415	Plane tree (wild strain) in Xiamafang Ruins Park, Nanjing City, Jiangsu Province
AC30	A154	Pagoda tree (wild strain) Jilin Agricultural University, Changchun City, Jilin Province
AC31	Ac31	Hybrid strain
AC32	A450	Sophora japonicus (wild strain), Chengzijie Town, Jiutai City, Jilin Province
AC33	L31	Sichuan Academy of Agricultural Sciences (Cultivated Strains)
AC34	Purple	Sichuan Academy of Agricultural Sciences (Cultivated Strains)
AC35	CR5	Zhangzhou Comprehensive Experimental Station (Cultivated Strains)
AC36	Zha10/	Zhangzhou Comprehensive Experimental Station (Cultivated Strains)
AC37	AY13	Sichuan Academy of Agricultural Sciences (Cultivated Strains)
AC38	A448	Acacia tree in Yitong, Jilin Province (wild strain)
AC39	A449	Acacia tree in Yitong, Jilin Province (wild strain)
AC40	Chuan Er 4	Sichuan Academy of Agricultural Sciences (Cultivated Strains)
AC41	Ac41	(wild strain) of Japan
AC42	Ac42	(wild strain) of Japan
AC43	Ac43	(wild strain) of Japan
AC44	Ac44	From Japan (Cultivated Strains)
AC45	Chuan Er 1	Sichuan Academy of Agricultural Sciences (Cultivated Strains)
AC46	781	Sichuan Academy of Agricultural Sciences (Cultivated Strains)
AC47	Yellow Ear. 10	Sichuan Academy of Agricultural Sciences (Cultivated Strains)
AC48	Ap6	Catalpa tree (wild strain), Jiaohe City, Jilin Province
AC49	Ap7	Catalpa tree (wild strain), Jiaohe City, Jilin Province
AC50	Ap15	Sichuan Academy of Agricultural Sciences (Cultivated Strains)

### Cultivation experiment

Mix the pre-wet sawdust substrate (78%) with wheat bran (20%), CaCO_3_ (1%), and CaSO_4_ (1%). Adjust the water content of the sawdust mixture to about 55 to 60%. Each polyethylene bag (height 30 cm, diameter 10 cm) was filled with 0.5 kg sawdust-based substrate, sterilized (121°C, 120 min), and then inoculated with 4 g/bag of prepared spawn. Inoculation bags were kept in the spawning chamber under dark conditions (temperature 25 ± 1°C, relative humidity 80%). When the mycelium was fully colonized, transfer the bags to the growing chamber (20 ± 2°C, relative humidity > 90%) to stimulate primordium formation. The fruiting bodies were then harvested by twisting method with clean hands for further analysis when fully grown to reveal waveform edge (16).

### Evaluation of agronomic traits

The agronomic characteristics enlisted as the time required for fruiting body formation and biological efficiency. The total number of days from inoculation bag to harvest fruiting bodies. Harvested fruiting bodies from the first and second flushes were weighed in fresh form for analysis of biological efficiency. Bioefficiency is the ratio of weight (g) of fresh fruiting body/dry weight of substrate (g), expressed as a percentage.

### Extraction of crude polysaccharides

According to the method proposed by Cai et al. (17) and Skalicka-Wozniak et al. (18), crude polysaccharides were extracted and purified from *A. cornea* strains and slightly modified. Five grams of dry powdered sample was extracted thrice with 200 mL hot water (80°C) for 3 hours. The water extract was filtered with fiber gauze, combined and then adjusted the volume up to 50 mL. Afterward, 150 mL of chilled ethanol (96%) was added and placed in a refrigerator at 4°C overnight to induce precipitation. After centrifugation (15,000 rpm, 6 min) the precipitate was collected, washed with ethanol, dried in the oven at 45°C and then grinded with a pestle and mortar for further analysis. The following equation has been used to measure the crude polysaccharide yield.

Yield (%) = *m*1/*m*2 × 100%, where *m*1 is the weight of crude polysaccharide, and *m*2 is the weight of *A. cornea* powder.

### Total polysaccharides

The total polysaccharide content in crude polysaccharides obtained from the fruiting body of the *A. cornea* strain was determined by phenol-sulfuric acid method (18). An equal weight of 1 mg sample of crude polysaccharide dissolved in 10 mL sterilized water. Then, mix 1 mL of this solution with 1 mL of phenol solution (5%) and 5 mL of concentrated sulfuric acid. The mixture was kept in the dark shaker at 25°C for 30 min, and measure the absorbance at 490 nm with a spectrophotometer. The polysaccharide content of *A. cornea* strains was determined by utilizing the glucose standard curve to calculate the total amount. We then set up nine glucose solutions from different sources, each with a different concentration of (1 mg·mL^−1^) glucose stock solution (i.e., (20, 40, 60, 80, 100, 120, 140, 160, and 180 μL) in a 10 mL centrifuge tube, adjust the volume to 2 mL by distilled water, then add 1 mL of 5% phenol solution and 5 mL of concentrated sulfuric acid, and repeat the same steps as above.

### Mineral quantification

The minerals present in the fruiting bodies of the *A. cornea* mushroom strain were measured using the method of (19) (cite the reference). The fruiting body samples are oven-dried at 35°C for 24 h and ground into a powder that can pass through a 1 mm sieve. Weighted 1 g of mushrooms in each sample to determine the mineral content. Afterward, “wet” digestion with a 3:1 mixture of nitric and perchloric acid. Finally, zinc, copper, manganese, and iron in solution were quantified using an atomic absorption spectrometer (19).

### Determination of antioxidant activity

DPPH radical scavenging using the method described by (20) was performed by vigorously mixing 2 mL of crude polysaccharide solution (0.25, 0.50, 0.75, 1.00, 1.25, and 1.50 mg/mL) and 2 mL of DPPH solution (0.2 mmol/L) and kept in the dark for 30 min and 2 mL of absolute ethanol solution was used as a control. Vitamin C is used as a standard and is formulated at the same concentration as crude polysaccharide solutions. A spectrophotometer is used to calculate absorbance at 517 nm. The following equation determines the free radical scavenging effect of DPPH:

Scavenging rate (%) = (Ablank − Asample)/Ablank × 100.

A blank represents the absorbance of the control solution (no sample), while A sample represents the absorbance of the test sample.

### ABTS radical scavenging assay

Free radical scavenging activity of crude polysaccharides was determined using the ABTS radical cation (ABTS+) test, a slightly modified Binsan’s method (17). The reaction of 7 mM ABTS solution with 2.45 mM potassium persulfate yields ABTS+, which was kept at room temperature for 16 h in the dark. At 734 mM, dilute the ABTS+ solution with ethanol to obtain an absorbance of 0.70 ± 0.02. Applied 1 mL of crude polysaccharide solution samples at different concentrations (0.25, 0.50, 0.75, 1.00, 1.25, and 1.50 mg/mL) to 3.9 mL of ABTS+ solution and mix vigorously. The absorbance at 734 nm was measured after 6 min of reaction at room temperature (17, 21).

### Statistical analysis

Data were analyzed using a well-known statistical method: Fisher Analysis of Variance (ANOVA). Treatment means were compared using the Least Significant Difference (LSD) test at the 5% probability level.

## Results and discussion

### Evaluation of agronomic traits

The biological efficiency of *A. cornea,* including the results of the fruiting bodies’ maturation period from the spawning stage to the point of harvest were described in Table 2. The shortest time of matured fruiting bodies of the *A. cornea* mushroom strain was recorded from wild strains Ac13 (80 days) and A24 (90 days). In comparison, the duration of the cultivated strain Ac44 was extended (166 days). The number of days of maturity of fruiting bodies of cultivated, wild and hybrid strains ranged from spawning to harvesting fruiting bodies (166–80 days).

**Table 2:** Total number of days for the maturity of fruiting bodies and biological efficiency of *A. cornea* strains.

Strains	Maturity of fruiting bodies in days	Biological efficiency	Strains	Maturity of fruiting bodies in days	Biological efficiency
AC1	93 ± 2^vw^	62.26 ± 1.6^cde^	AC26	105 ± 7^pqrstu^	16.57 ± 3.3^vwxy^
AC2	103 ± 4^qrstu^	63.22 ± 4.1^cde^	AC27	145 ± 6^cd^	0.986 ± 0.2
AC3	99 ± 5^stuvw^	82.40 ± 4.2^b^	AC28	105 ± 4^pqrstu^	17.59 ± 2.6^uvwx^
AC4	110 ± 8^nopqr^	20.30 ± 1.1^stuvwx^	AC29	116 ± 4^klmno^	7.946 ± 0.5^yz^
AC5	115 ± 4^lmnop^	14.69 ± 1.3^wxyz^	AC30	126 ± 5^fghij^	32.73 ± 2.6^klmno^
AC6	121 ± 4^ijklm^	7.400 ± 0.4^z^	AC31	104 ± 3^qrstu^	35.92 ± 0.3^jklmn^
AC7	155 ± 2^bc^	2.333 ± 0.3	AC32	121 ± 4^ijklm^	6.946 ± 0.8^z^
AC8	102 ± 2^rstuv^	20.80 ± 0.7^rstuvwx^	AC33	103 ± 3^rstuv^	29.09 ± 3.6^nopqrs^
AC9	113 ± 4^mnopq^	2.333 ± 0.2	AC34	156 ± 3^ab^	23.60 ± 3.1^pqrstuv^
AC10	154 ± 6^bc^	1.426 ± 0.1	AC35	118 ± 5^jklmn^	19.82 ± 7.1^tuvwx^
AC11	98 ± 103^tuvw^	63.73 ± 5.1^cde^	AC36	110 ± 4^monpr^	48.66 ± 6.7^fgh^
AC12	121 ± 3^hijklm^	29.02 ± 2.4^nopqrs^	AC37	126 ± 4^fghijk^	65.06 ± 2.7^cd^
AC13	80 ± 3^x^	29.85 ± 1.8^lmnopq^	AC38	131 ± 8^fghi^	22.24 ± 5.2^qrstuvw^
AC14	108 ± 5^opqrst^	28.60 ± 3.3^nopqrst^	AC39	134 ± 7^ef^	8.013 ± 1.6^yz^
AC15	105 ± 11^pqrstu^	94.84 ± 5.3^a^	AC40	100 ± 2^rstuv^	70.27 ± 4.7^c^
AC16	103 ± 5^rstu^	37.12 ± 0.8^ijklmno^	AC41	136 ± 4^def^	16.96 ± 3.7^uvwx^
AC17	142 ± 7^de^	38.16 ± 2.2^ijklm^	AC42	126 ± 6^fghijk^	7.106 ± 1.6^z^
AC18	116 ± 6^klmno^	18.29 ± 1.9^uvwx^	AC43	131 ± 4^fgh^	21.47 ± 3.5^qrstuvwx^
AC19	123 ± 4^ghijkl^	25.63 ± 3.8^opqrstu^	AC44	166 ± 4^a^	57.33 ± 11.8^def^
AC20	161 ± 8^ab^	17.77 ± 2.6^uvwx^	AC45	106 ± 5^pqrstu^	55.48 ± 8.1^ef^
AC21	104 ± 4^qrstu^	13.09 ± 1.2^xyz^	AC46	109 ± 4^nopqrs^	29.43 ± 6.8^mnopqr^
AC22	132 ± 8^fg^	28.67 ± 0.6^nopqrst^	AC47	109 ± 6^nopqr^	32.27 ± 3.2^klmop^
AC23	97 ± 5^uvw^	38.61 ± 3.6^ijkl^	AC48	101 ± 2^rstuv^	13.73 ± 1.7^wxyz^
AC24	90 ± 3^wx^	52.95 ± 4.2^fg^	AC49	118 ± 9^jklmn^	39.33 ± 0.8^ijk^
AC25	107 ± 98^opqrstu^	42.03 ± 1.8^hij^	AC50	103 ± 6^qrstu^	45.86 ± 7.5^ghi^

Values with no letter in common in each column are significantly different (*p* < 0.05) (means ± SD, *n* = 3).

The time to complete fruiting bodies recorded from cultivated *A. cornea* strains was more than 90 days and longer for wild strains compared to cultivated strains (16). This study reports the attainment of the shortest duration for the completion of fruiting bodies from wild and domesticated strains, representing a novel finding. (i.e., Ac13 and Ac24). The variances in biological efficiency among *A. cornea* strains, including cultivated, wild, and hybrid varieties, have been observed. The hybrid strain Ac15 showed the highest biological efficiency (94.84%), while the wild strain Ac27 indicted the lowest biological efficiency (0.92%). The biological efficiency of *A. cornea* strains ranged from (94.84–0.986%) as shown in Table 2.

### Crude polysaccharide content

**Figure 1:**
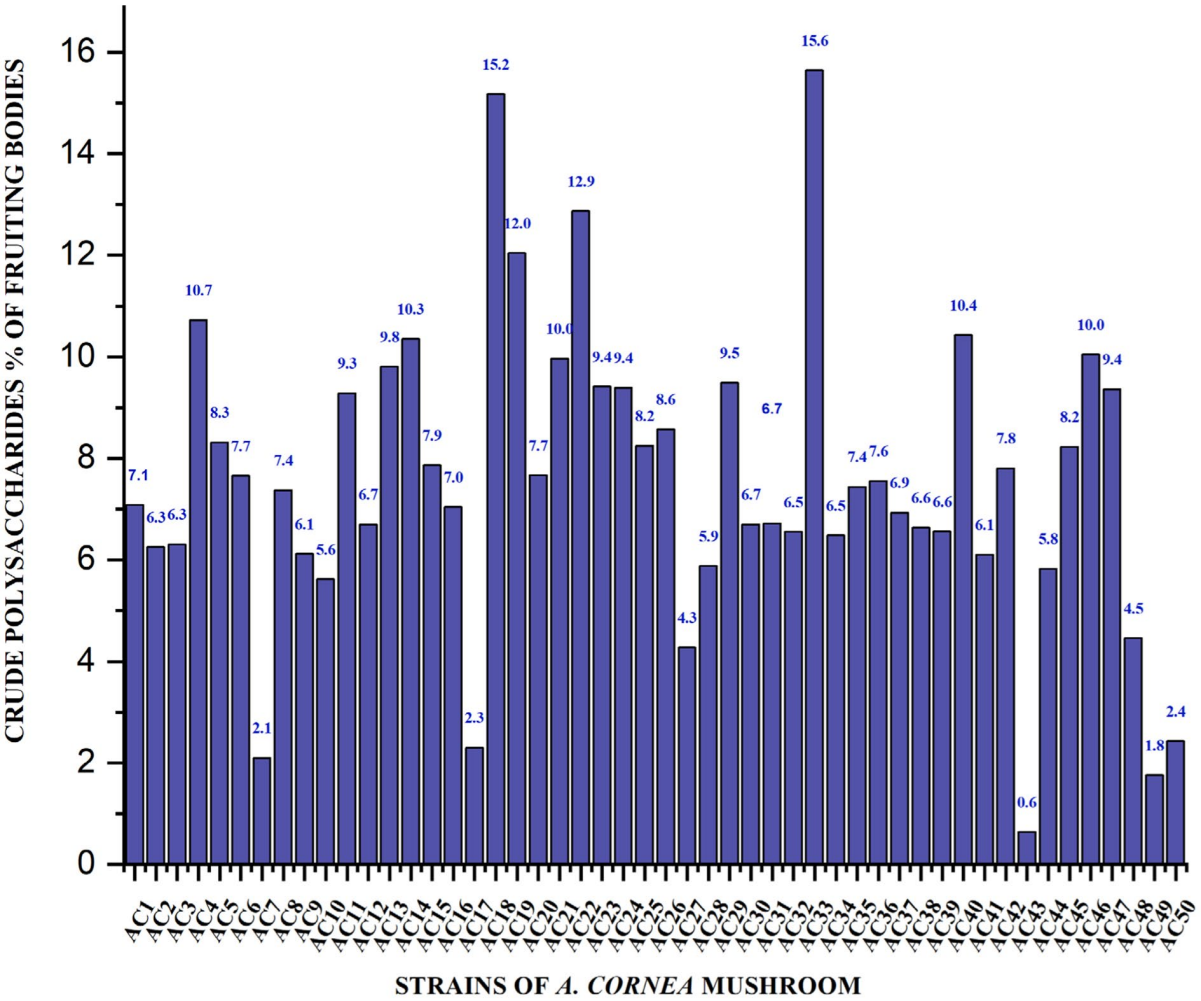
Crude polysaccharides percentage of fruiting bodies of *A. cornea* strains.

The crude polysaccharide content of cultivated, wild and hybrid *A. cornea* strain was thoroughly investigated. Crude polysaccharides extracted from the fruiting bodies of the *A. cornea* strain range from 15.64–0.63% (Figure 1). The cultivated *A. cornea* strain Ac33 demonstrated the highest crude polysaccharide content (15.64%). While the Japanese wild strain Ac43 indicted the lowest crude polysaccharide content (0.63%). In addition, the outcomes obtained from the crude polysaccharides derived from the hybrid strain Ac33 were found to be twice as much as the control utilized by Li et al. Specifically, the control group exhibited a dry weight of 7%, whereas the selenium supplementation in the control group resulted in a 23% dry weight (14).

### Total polysaccharide content

**Figure 2:**
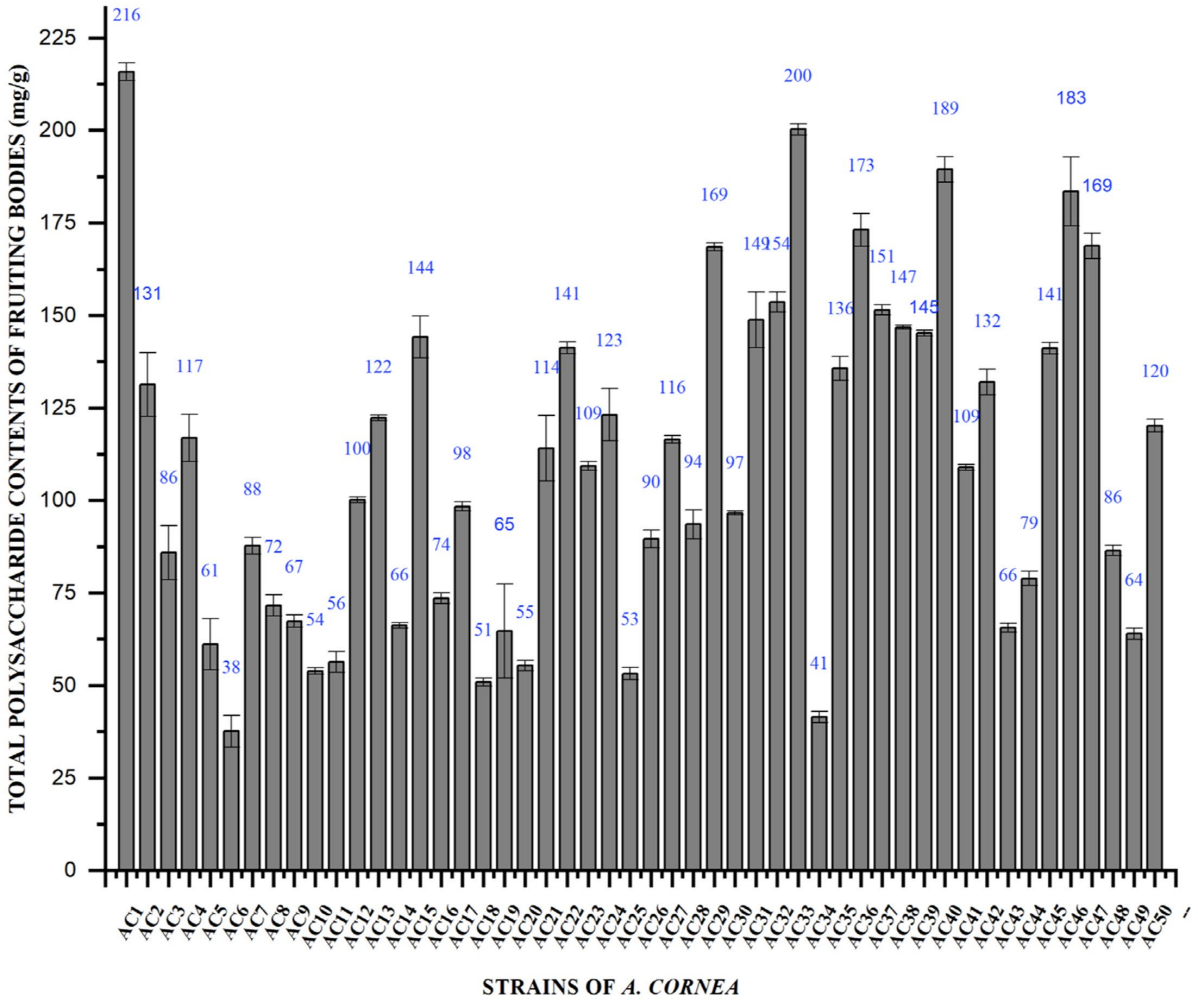
Total polysaccharide contents of fruiting bodies of *A. cornea* strains.

Total polysaccharide content of cultivated, wild and hybrid strains of *A. cornea* fruiting bodies was determined by the phenol-sulfuric acid method and the results were shown in Figure 2. The total polysaccharide content of fruiting bodies of the *A. cornea* mushroom strain varied considerably, from 215.88 ± 2.4 mg. g^−1^ to 37.63 ± 4.3 mg. g^−1^. The highest contents of total polysaccharides were obtained from cultivated strained Ac1 (215.88 mg. g^−1^). The lowest total polysaccharide content of fruiting bodies was observed in the cultivated strain Ac6 (37.63 mg. g^−1^). The study conducted by Su et al. revealed that the Auricularia species exhibited a range of 71.3 to 81.49 g in total polysaccharide content. The outcome of 100 g^−1^ is comparable to these results (22).

### Mineral content

The mineral composition of the fruiting body of domesticated, wild, and hybrid *A. cornea* strains are shown in Table 3. The copper and manganese content of the *A. cornea* was 0.133 to 8.40 mg. kg^−1^ and 213 to 788 mg. kg^−1^, respectively. The cultivated strain Ac46 showed the highest copper content (8.40 mg. kg^−1^), followed by wild strain Ac49 (8.36 mg. kg^−1^) and hybrid strain Ac19 (8.2 mg. kg^−1^), while wild strain Ac29 contained the lowest copper content (0.133 mg. kg^−1^).

**Table 3:** Mineral contents of *A. cornea* strains.

Strains	Copper (mg/kg)	Manganese (mg/kg)	Zinc (mg/kg)	Iron (mg/kg)	Strains	Copper (mg/kg)	Manganese (mg/kg)	Zinc (mg/kg)	Iron (mg/kg)
AC1	7.200 ± 0.1^bc^	483.33 ± 5^fg^	318.00 ± 13^mnopqrs^	229.00 ± 5.1^hi^	AC26	0.700 ± 0.1^wxyz^	276.00 ± 18^o^	417.7 ± 33^bcdefg^	187 ± 8.6^mn^
AC2	2.366 ± 0.2^opqr^	375.33 ± 8^j^	391.67 ± 35^cdefghij^	92.333 ± 3.4^qrs^	AC27	1.300 ± 0.2^tuvwx^	774.00 ± 11^a^	243.7 ± 27^tuvw^	343.7 ± 8.0^ab^
AC3	4.266 ± 0.3^ijkl^	788.33 ± 19^a^	215.00 ± 12^vwx^	77.333 ± 3.3^rstu^	AC28	0.966 ± 0.2^vwxyz^	340.00 ± 12^m^	169.3 ± 14^x^	350 ± 27.5^a^
AC4	1.400 ± 0.2^tuvw^	340.67 ± 16^m^	197.00 ± 6^wx^	96.333 ± 5.2^pqr^	AC29	0.133 ± 0.0^z^	669.00 ± 11^b^	357.7 ± 27^ghijklmno^	332 ± 7.8^abc^
AC5	2.76 ± 0.7^mnopq^	462.00 ± 27^gh^	326.33 ± 33^klmnopq^	200.00 ± 2.2^klm^	AC30	1.833 ± 0.5^rstu^	670.00 ± 13^b^	376 ± 45^efghijklm^	299.3 ± 1.2^e^
AC6	1.833 ± 0.2^rstu^	543.67 ± 15^de^	232.33 ± 39^uvwx^	177.00 ± 8.2^no^	AC31	6.733 ± 0.2^cd^	457.67 ± 7^h^	433.3 ± 23^abcde^	95 ± 3.3^qrs^
AC7	3.033 ± 0.2^mnop^	366.67 ± 17^jkl^	285.67 ± 44^qrstu^	245.67 ± 30.9^gh^	AC32	3.466 ± 0.3^lm^	381.00 ± 13^j^	436 ± 13^abcde^	159 ± 30.7^o^
AC8	4.400 ± 0.2^ijk^	207.00 ± 4^p^	174.33 ± 45^x^	220.33 ± 8.2^ijk^	AC33	2.90 ± 0.6^mnopq^	766.00 ± 11^a^	354.7 ± 34^ghijklmno^	304.3 ± 3.4^de^
AC9	1.500 ± 0.3^stuvw^	566.33 ± 19^cd^	316.00 ± 25^mnopqrs^	68.667 ± 2.1^tu^	AC34	7.700 ± 0.4^ab^	458.00 ± 4^h^	254.67 ± 17^stuvw^	81 ± 5.7^rst^
AC10	6.300 ± 0.2^de^	301.33 ± 9^n^	244.33 ± 59^tuvw^	58.000 ± 2.9^u^	AC35	5.100 ± 1.3^ghi^	226.00 ± 4^p^	361.0 ± 43^fghijklmno^	65.03 ± 4.2^tu^
AC11	0.500 ± 0.2^xyz^	497.33 ± 14^f^	313.33 ± 8^mnopqrs^	92.333 ± 5.0^qrs^	AC36	3.233 ± 1.0^mn^	575.67 ± 9^c^	328.3 ± 10^jklmnopq^	27 ± 4.3^v^
AC12	4.933 ± 0.4^ghij^	343.67 ± 9l^m^	423.67 ± 6^abcdef^	67.667 ± 2.5^tu^	AC37	4.266 ± 0.4^ijkl^	672.33 ± 5^b^	357 ± 45^ghijklmno^	87 ± 2.8^qrst^
AC13	4.366 ± 0.4^ijk^	295.33 ± 6^n^	387.33 ± 18^defghijk^	75.000 ± 2.2^rstu^	AC38	3.200 ± 0.9^mno^	340.33 ± 5^m^	408.33 ± 47^bcdefgh^	197 ± 3.7^lmn^
AC14	7.200 ± 0.3^bc^	428.00 ± 23^i^	440.00 ± 11^abcd^	97.00 ± 0.8^pqr^	AC39	0.366 ± 0.2^yz^	561.67 ± 5^cd^	350 ± 28^hijklmnop^	204 ± 5.6^jklm^
AC15	2.433 ± 0.2^nopqr^	380.00 ± 7^j^	291.00 ± 21^pqrstu^	275.67 ± 15.9^f^	AC40	1.100 ± 0.2^uvwxy^	778.33 ± 13^a^	310.3 ± 58^nopqrs^	311 ± 8.2^cde^
AC16	6.166 ± 0.2^def^	536.00 ± 20^e^	280.67 ± 50^qrstu^	187.67 ± 8.2^mn^	AC41	1.466 ± 0.2^stuvw^	569.00 ± 5^c^	360.3 ± 19^fghijklmno^	257.7 ± 17.9^fg^
AC17	3.333 ± 0.3^m^	440.33 ± 11^hi^	350.67 ± 27^hijklmnop^	93.333 ± 2.6^qrs^	AC42	3.600 ± 0.4^klm^	448.0 ± 11^hi^	460 ± 32^ab^	332.3 ± 9.0^abc^
AC18	5.366 ± 0.4^fgh^	667.67 ± 10^b^	383.33 ± 39^defghijkl^	217.7 ± 12.5^ijkl^	AC43	0.700 ± 0.4^wxyz^	771.33 ± 7^a^	454 ± 32^abc^	216.3 ± 4.2^ijkl^
AC19	8.200 ± 0.4^a^	338.67 ± 13^m^	285.33 ± 7^qrstu^	72.667 ± 5.6^stu^	AC44	2.300 ± 0.3^pqrs^	455.33 ± 8^h^	366 ± ^48fghijklmn^	304.3 ± 12.5^de^
AC20	4.233 ± 0.8^jkl^	213.33 ± 4^p^	300.33 ± 1^opqrst^	185.67 ± 10.7^mn^	AC45	6.500 ± 0.3^cde^	770.00 ± 4^a^	369.3 ± 34^fghijklmn^	108.7 ± 6.1^pq^
AC21	4.866 ± 0.2^hij^	543.67 ± 19^de^	246.33 ± 29^tuvw^	221.33 ± 7.1^ijk^	AC46	8.400 ± 0.4^a^	347.6 ± 10^klm^	486.33 ± 40^a^	229.3 ± 26.6^hi^
AC22	6.333 ± 0.3^de^	658.67 ± 12^b^	400.33 ± 1^bcdefghi^	325.0 ± 12.6^bcd^	AC47	1.700 ± 0.4^rstuv^	453.67 ± 2^h^	402.33 ± 58^bcdefghi^	318.3 ± 5.2^cde^
AC23	1.16 ± 0.1^uvwxy^	452.33 ± 17^hi^	341.67 ± 13^ijklmnopq^	318.67 ± 3.3^cde^	AC48	5.766 ± 0.5^efg^	560.33 ± 5^cde^	320.33 ± 18l^mnopqr^	322 ± 8.6^bcd^
AC24	1.266 ± 0.2^tuvwx^	371.00 ± 25^jk^	261.67 ± 12^rstuv^	310.67 ± 6.0^cde^	AC49	8.366 ± 0.5^a^	676.00 ± 1^b^	354.7 ± 15^ghijklmno^	18,833 ± 5.2^p^
AC25	2.100 ± 0.2^qrst^	560.3 ± 15^cde^	373.0 ± 57^efghijklmn^	224.67 ± 12.4^hij^	AC50	6.666 ± 0.4^cd^	774.00 ± 10^a^	435.33 ± 13^abcde^	229 ± 18.2^hi^

Values with no letter in common in each column are significantly different (*p* < 0.05) (means ± SD, *n* = 3).

Significant differences were observed in the manganese content of *A. cornea* strains, with the highest manganese content in hybrid strains Ac3 (788 mg. kg^−1^), followed by cultivated strain Ac40 (778 mg. kg^−1^), and then wild strain Ac27 (774 mg. kg^−1^). In contrast, the hybrid strain Ac20 showed the lowest (213 mg. kg^−1^). Among the 50 *A. cornea* stains, the cultivated strain Ac46 showed the highest zinc content (486.33 mg. kg^−1^). At the same time, the wild strain Ac28 demonstrated the highest iron content (350 mg. kg^−1^). In contrast, the wild strain Ac28 contained the lowest zinc content (169 mg. kg^−1^), while the cultivated strain Ac36 showed the lowest iron content (27 mg. kg^−1^). In general, *A. cornea* was high in zinc and iron contents. Mushrooms are good natural zinc and iron accumulators and biologically essential for the human body. Our findings are consistent with the results of Wang et al., who investigated the mineral content of various *A. cornea* strains. In addition, prior study of Rebecca et al., showed lower results for iron, manganese, copper, and iron contents compare to our results (15).

In the current study, we found that *A. cornea* mushrooms can be used in various food items. Malnutrition is currently causing health issues for people all over the world. It is estimated that 17% of the population is at risk of zinc deficiency (12). This decline is due to insufficient levels of these elements (copper, manganese, zinc, and iron) in a healthy diet. Implementing effective strategies to prevent and regulate these nutrients in the human diet is therefore essentially required. Hence, there is a trend of increasing dietary supplements worldwide. While the increase in their chemical composition requires a natural process of absorption and accumulation, mushrooms are screened for their high nutritional and commercial value (23). The *A. cornea* mushrooms are considered to be a significant source of vital nutrients. Thus, the utilization of *A. cornea* mushrooms has the potential to efficiently mitigate nutritional insufficiencies prevalent in impoverished and malnourished populations. Furthermore, these findings establish the basis for novel germplasm advancements.

### DPPH radical scavenging activity

**Figure 3:**
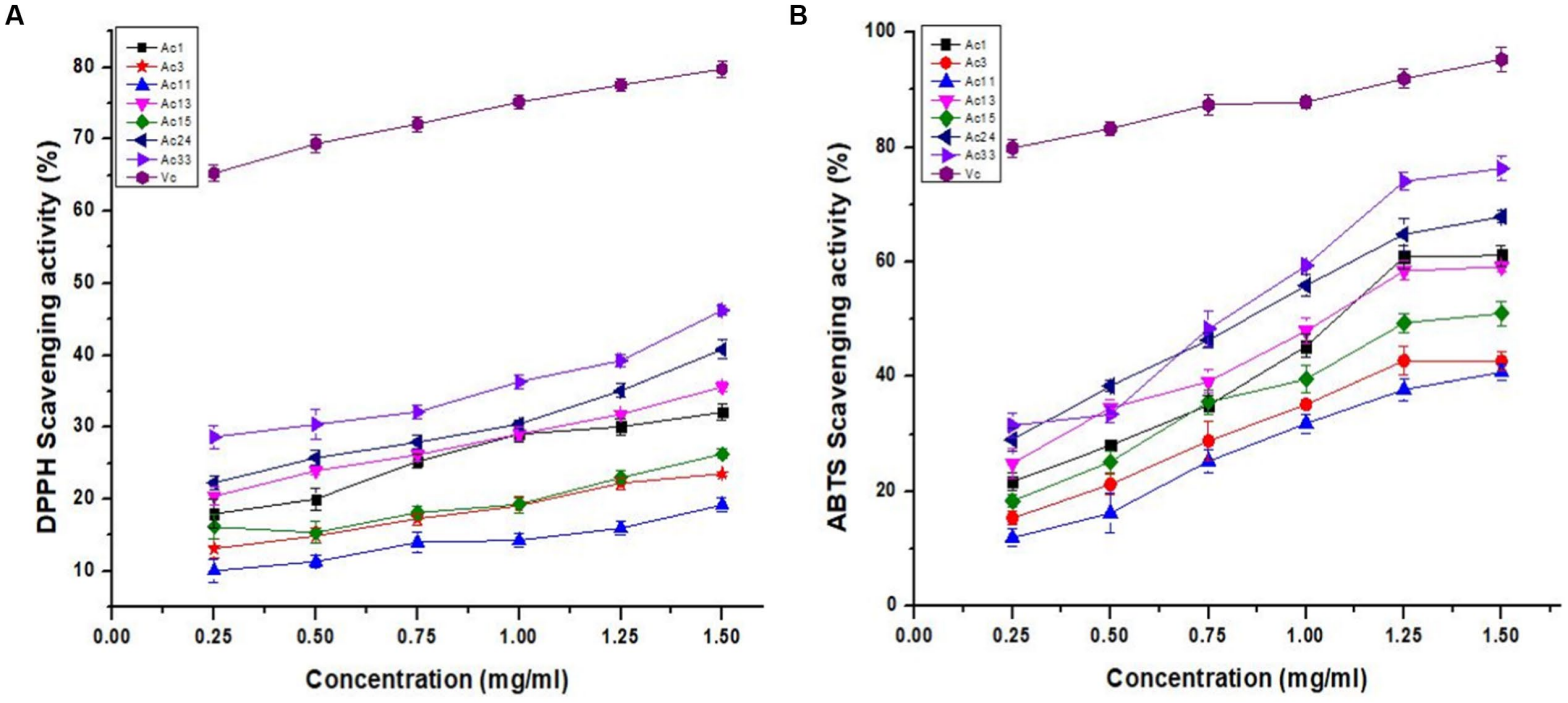
Antioxidant activities of crude polysaccharides of *A. cornea* strains. **(A)** DPPH radical scavenging and **(B)** ABTS scavenging activities.

DPPH radicals are static and are commonly used to assess the radical scavenging activity of biological compounds. Biological compounds can transfer an electron or a hydrogen atom to a DPPH radical, which is how they scavenge DPPH radical (24). This is a commonly used technique to determine the sensitivity, incompetence, and velocity of many samples to determine their antioxidant capacity (25). As shown in Figure 3A, the DPPH radical scavenging capacity of all crude polysaccharide *A. cornea* has a concentration-dependent connection. The concentration-dependent DPPH radical scavenging potential of strains of *A. cornea* is demonstrated in Figure 3A. When the concentration of crude polysaccharides increased from 0.25–1.50 mg/mL, the scavenging activity of crude polysaccharides on DPPH free radicals progressively increased. The highest scavenging rate appears at a crude polysaccharide concentration of 1.50 mg/mL. The strongest scavenging rates of Ac1, Ac3, Ac11, Ac13, Ac15, Ac24, and Ac33 were 32.1, 23.6, 19.2, 35.6, 26.3, 40.8, and 46.2%, respectively, weaker than Vc (vitamin C). Seven types of *A.cornea* mushroom strains crude polysaccharides scavenge free radicals: Ac33 > Ac24 > Ac13 > Ac1 > Ac15 > Ac3 > Ac11. The scavenging activity on DPPH radicles was similar to that of crude polysaccharides from *Lepista nuda,* and was relatively smaller than that of polysaccharides from *Auricularia* species (10, 22, 26).

### ABTS radical scavenging activity

ABTS radical scavenging activity is a simple and commonly used procedure for measuring the antioxidant activity of natural ingredients. As shown in Figure 3B, crude polysaccharides from 7 *A. cornea* strains showed 11.9–76.3% ABTS radical scavenging activity at concentrations of 0.25–1.50 mg/mL. It was found that ABTS radical scavenging activity increased with increasing crude polysaccharide concentration. The crude polysaccharides of commercially cultivated strain Ac33 achieved 76.3% maximum ABTS radical scavenging activity at 1.50 mg/mL, while the hybrid strain Ac11 obtained 11.9% minimum ABTS radical scavenging activity at 0.25 mg/mL. However, at a maximum concentration of 1.50 mg/mL, standard vitamin C (Vc) had 95.3% ABTS radical scavenging activity. The potential of seven crude polysaccharides to scavenge ABTS free radicals was Ac33 > Ac24 > Ac1 > Ac13 > Ac15 > Ac3 > Ac11. Similarly, the polysaccharides obtained from *Pleurotus sajor* caju showed ABTS radical scavenging capacity of 16.01 to 70.09% at different 25 to125 μg/mL concentrations. In comparison, polysaccharides from *Pleurotus sajor caju* showed an ABTS scavenging capacity of 63.96% at 5 mg/mL concentrations (27). Similarly, at a 10 mg/mL concentration, the ABTS clearance efficiency of melanin extracted from black fungus was 95.6%29. Our results correlate significantly with previous work (28).

### Principal components analysis

**Figure 4:**
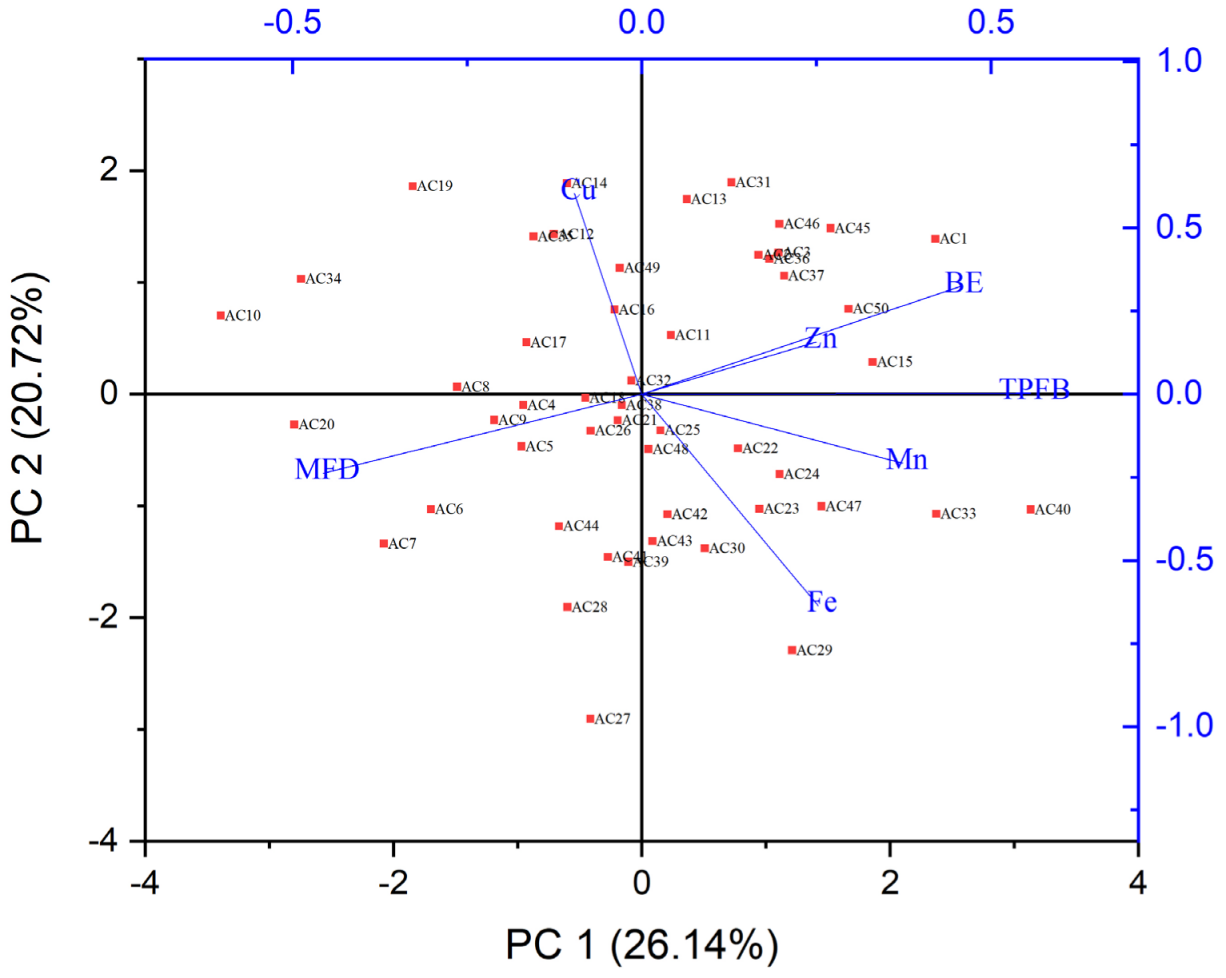
Biplot based on Principal Component Analysis of mushroom chemical composition and *A. cornea* strains arrangement. (Ac), mushroom strains; (MFD), numbers of days for harvesting of fruiting bodies; (BE), biological efficiency; (Zn), zinc; (Fe), iron; (Cu), copper; (Mn), manganese, and (TPB), total polysaccharides of fruiting bodies.

The differences and similarities between the chemical compounds and agronomic traits of *A. cornea* mushroom strains were subjected to principal component analysis (PCA) as shown in Figure 4. The study involved the analysis of six distinct components, namely the total number of days required for harvesting of fruiting bodies and biological efficiency, across a sample of 50 strains of *A. cornea*. Principal Component Analysis (PCA) is a commonly employed technique aimed at reducing a large number of variables to a limited set of principal components. These components are selected based on their ability to account for the maximum variance present in the data being analyzed. The distributions of all the samples are conveniently positioned around the center of the map, as shown by the results. The first two principal components (PC1 and PC2) accounted for 46.86% of the total variance (26.14 and 20.72%, respectively). The PC1 was associated with the contents of iron, zinc, manganese, total polysaccharides of fruiting bodies, biological efficiency whereas the PC2 was correlated with MFD and Copper. The components were closed to each other, positively correlated, such as iron, manganese, the total polysaccharides of fruiting bodies.

On the other hand, certain compounds such as zinc, crude polysaccharides and biological efficiency were not significantly correlated with iron, manganese, the total polysaccharides of fruiting bodies, but they were closely significant with each other. Some compounds were separated, they were negatively correlated, such as copper and days to maturity of fruiting bodies. The principal component analysis (PCA) results elucidated the interrelationships among different compounds and explicated their distinct and affirmative correlations.

## Conclusion

The study determined that the crude polysaccharides derived from various strains of *A. cornea* mushrooms exhibit natural antioxidant properties. Additionally, the investigation revealed that wild, hybrid, and commercial strains of *A. cornea* mushrooms are capable of attaining accelerated growth, premature maturation, and elevated productivity. The scientific basis for commencing high-quality breeding and producing “functional food” with genuine nutritional and health value, as well as the development of innovative antioxidant food additives and active ingredients of different fungal strains, was provided by the assessment of biochemical indexes and nutritional characteristics of strains with exceptional traits. Additionally, the germplasm resources were established for this purpose, along with abundant resources of crude polysaccharides.

## Data availability statement

The original contributions presented in the study are included in the article/supplementary material, further inquiries can be directed to the corresponding authors.

## Author contributions

AK, F-JY, MF, L-XL, and Y-MZ: conceptualization. PW and J-JM: methodology. AK and BX: writing and review. F-JY, MF, L-XL, and Y-hW: supervision. K-SS, X-XM, and QH: analysis. All authors contributed to the article and approved the submitted version.

## Funding

The research was supported by China Agriculture Research System (No. CARS-20) and The project of “one thousand talents plan” in Jiangxi Province, China.

## Conflict of interest

The authors declare that the research was conducted in the absence of any commercial or financial relationships that could be construed as a potential conflict of interest.

## Publisher’s note

All claims expressed in this article are solely those of the authors and do not necessarily represent those of their affiliated organizations, or those of the publisher, the editors and the reviewers. Any product that may be evaluated in this article, or claim that may be made by its manufacturer, is not guaranteed or endorsed by the publisher.

## References

[1] Yang K Zhang S Ying Y Li Y Cai M Guan R. Cultivated fruit body of Phellinus baumii: a potentially sustainable antidiabetic resource. ACS Omega. (2020) 5:8596–604. doi: 10.1021/acsomega.9b04478, PMID: 32337422 PMC7178366

[2] Zeng W-C Zhang Z Gao H Jia L-R Chen W-Y. Characterization of antioxidant polysaccharides from Auricularia auricular using microwave-assisted extraction. Carbohydr Polym. (2012) 89:694–700. doi: 10.1016/j.carbpol.2012.03.078, PMID: 24750775

[3] Thu ZM Myo KK Aung HT Clericuzio M Armijos C Vidari G. Bioactive phytochemical constituents of wild edible mushrooms from Southeast Asia. Molecules. (2020) 25:1972. doi: 10.3390/molecules25081972, PMID: 32340227 PMC7221775

[4] Deveci E Çayan F Tel-Çayan G Duru ME. Structural characterization and determination of biological activities for different polysaccharides extracted from tree mushroom species. J Food Biochem. (2019) 43:e12965. doi: 10.1111/jfbc.12965, PMID: 31489667

[5] Ma F Wu J Li P Tao D Zhao H Zhang B. Effect of solution plasma process with hydrogen peroxide on the degradation of water-soluble polysaccharide from Auricularia auricula. II: solution conformation and antioxidant activities in vitro. Carbohydr Polym. (2018) 198:575–80. doi: 10.1016/j.carbpol.2018.06.113, PMID: 30093036

[6] Miao J Regenstein JM Qiu J Zhang J Zhang X Li H. Isolation, structural characterization and bioactivities of polysaccharides and its derivatives from Auricularia-a review. Int J Biol Macromol. (2020) 150:102–13. doi: 10.1016/j.ijbiomac.2020.02.054, PMID: 32044370

[7] Matkowski A Tasarz P Szypuła E. Antioxidant activity of herb extracts from five medicinal plants from Lamiaceae, subfamily Lamioideae. J Med Plants Res. (2008) 2:321–30.

[8] Yuan J-F Zhang Z-Q Fan Z-C Yang J-X. Antioxidant effects and cytotoxicity of three purified polysaccharides from Ligusticum chuanxiong Hort. Carbohydr Polym. (2008) 74:822–7. doi: 10.1016/j.carbpol.2008.04.040

[9] Shi M Zhang Z Yang Y. Antioxidant and immunoregulatory activity of Ganoderma lucidum polysaccharide (GLP). Carbohydr Polym. (2013) 95:200–6. doi: 10.1016/j.carbpol.2013.02.081, PMID: 23618260

[10] Chen Y Xue Y. Purification, chemical characterization and antioxidant activities of a novel polysaccharide from Auricularia polytricha. Int J Biol Macromol. (2018) 120:1087–92. doi: 10.1016/j.ijbiomac.2018.08.160, PMID: 30170055

[11] Khan AA Yao F Idrees M Lu L Fang M Wang P. A comparative study of growth, biological efficiency, antioxidant activity and molecular structure in wild and commercially cultivated Auricularia cornea strains. Folia Horticulturae. (2020) 32:255–64. doi: 10.2478/fhort-2020-0023

[12] Rzymski P Mleczek M Niedzielski P Siwulski M Gąsecka M. Cultivation of Agaricus bisporus enriched with selenium, zinc and copper. J Sci Food Agric. (2017) 97:923–8. doi: 10.1002/jsfa.7816, PMID: 27218432

[13] Wang P Yao F-J Lu L-X Fang M Zhang Y-M Khan AA. Map-based cloning of genes encoding key enzymes for pigment synthesis in Auricularia cornea. Fungal Biol. (2019) 123:843–53. doi: 10.1016/j.funbio.2019.09.002, PMID: 31627860

[14] Li X Yan L Li Q Tan H Zhou J Miao R. Transcriptional profiling of Auricularia cornea in selenium accumulation. Sci Rep. (2019) 9:1–13. doi: 10.1038/s41598-019-42157-2 30948778 PMC6449350

[15] Rebecca R Zhang J Zhao W Liu Y Wasantha K Wang S. Compositional and nutritional inventory of naturally mutant strain Auricularia corneavar Li edible mushroom from China. Prog Nutr. (2020) 22:323–9.

[16] Liang C-H Wu C-Y Lu P-L Kuo Y-C Liang Z-C. Biological efficiency and nutritional value of the culinary-medicinal mushroom Auricularia cultivated on a sawdust basal substrate supplement with different proportions of grass plants. Saudi J Biol Sci. (2019) 26:263–9. doi: 10.1016/j.sjbs.2016.10.017, PMID: 31485164 PMC6717086

[17] Cai M Lin Y Luo Y-L Liang H-H Sun P. Extraction, antimicrobial, and antioxidant activities of crude polysaccharides from the wood ear medicinal mushroom Auricularia auricula-judae (higher basidiomycetes). Int J Med Mushrooms. (2015) 17:591–600. doi: 10.1615/IntJMedMushrooms.v17.i6.90, PMID: 26349516

[18] Skalicka-Wozniak K Szypowski J Los R Siwulski M Sobieralski K Glowniak K. Evaluation of polysaccharides content in fruit bodies and their antimicrobial activity of four Ganoderma lucidum (W Curt.: Fr.) P Karst strains cultivated on different wood type substrates. Acta Soc Botanicorum Poloniae. (2012) 81:17–21. doi: 10.5586/asbp.2012.001

[19] Khan AA Muhammad MJ Muhammad I Jan I Samin G Zahid A. Modulation of agronomic and nutritional response of pleurotus eryngii strains by utilizing glycine betaine enriched cotton waste. J Sci Food Agric. (2019) 99:6911–21. doi: 10.1002/jsfa.9977, PMID: 31393604

[20] Xiao H Fu X Cao C Li C Chen C Huang Q. Sulfated modification, characterization, antioxidant and hypoglycemic activities of polysaccharides from Sargassum pallidum. Int J Biol Macromol. (2019) 121:407–14. doi: 10.1016/j.ijbiomac.2018.09.197, PMID: 30291933

[21] Liu J Li Y Liu W Qi Q Hu X Li S. Extraction of polysaccharide from Dendrobium nobile Lindl. By subcritical water extraction. ACS Omega. (2019) 4:20586–94. doi: 10.1021/acsomega.9b02550, PMID: 31858044 PMC6906767

[22] Su Y Li L. Structural characterization and antioxidant activity of polysaccharide from four auriculariales. Carbohydr Polym. (2020) 229:115407. doi: 10.1016/j.carbpol.2019.115407, PMID: 31826485

[23] Pastori GM Kiddle G Antoniw J Bernard S Veljovic-Jovanovic S Verrier PJ. Leaf vitamin C contents modulate plant defense transcripts and regulate genes that control development through hormone signaling. Plant Cell. (2003) 15:939–51. doi: 10.1105/tpc.010538, PMID: 12671089 PMC152340

[24] Naik G Priyadarsini K Satav J Banavalikar M Sohoni D Biyani M. Comparative antioxidant activity of individual herbal components used in ayurvedic medicine. Phytochemistry. (2003) 63:97–104. doi: 10.1016/S0031-9422(02)00754-9, PMID: 12657303

[25] Ma Y He H Wu J Wang C Chao K Huang Q. Assessment of polysaccharides from mycelia of genus Ganoderma by mid-infrared and near-infrared spectroscopy. Sci Rep. (2018) 8:1–10. doi: 10.1038/s41598-017-18422-7 29311571 PMC5758644

[26] Shu X Zhang Y Jia J Ren X Wang Y. Extraction, purification, and properties of water-soluble polysaccharides from mushroom Lepista nuda. Int J Biol Macromol. (2019) 128:858–69. doi: 10.1016/j.ijbiomac.2019.01.214 30716376

[27] Seedevi P Ganesan AR Mohan K Raguraman V Sivakumar M Sivasankar P. Chemical structure and biological properties of a polysaccharide isolated from Pleurotus sajor-caju. RSC Adv. (2019) 9:20472–82. doi: 10.1039/C9RA02977J, PMID: 35514737 PMC9065548

[28] Liu X Hou R Wang D Mai M Wu X Zheng M. Comprehensive utilization of edible mushroom Auricularia auricula waste residue—extraction, physicochemical properties of melanin and its antioxidant activity. Food Sci Nutr. (2019) 7:3774–83. doi: 10.1002/fsn3.1239, PMID: 31763027 PMC6848827

